# Cancer-associated fibroblasts-derived exosomal circ_0067557 promotes colorectal cancer epithelial-mesenchymal transition via BHLHE40-mediated transcriptional activation of OTUB2

**DOI:** 10.1186/s13062-026-00805-4

**Published:** 2026-05-11

**Authors:** Daoxu Zhang, Jiaqi Zhang, Cheng Yang, Yan Wang, Muhong Wang, Zhiwei Yu

**Affiliations:** 1 Department of Colorectal Surgery, Harbin Medical University Cancer Hospital, No. 150, Haping Road, Nangang District, Harbin City, Heilongjiang Province 150086 China; 2 Department of Chest Radiotherapy, Harbin Medical University Cancer Hospital, Harbin, 150086 China

**Keywords:** Colorectal cancer, Exosome, circ_0067557, BHLHE40, OTUB2, Epithelial-mesenchymal transition

## Abstract

**Objective:**

This study aimed to clarify how cancer-associated fibroblast-derived exosomal circ_0067557 (CAF-exo circ_0067557) promotes epithelial-mesenchymal transition (EMT) in colorectal cancer (CRC) through BHLHE40-mediated transcriptional activation of OTUB2.

**Methods:**

CAF-derived exosomes were isolated and characterized from CRC tissues, while normal fibroblast-derived exosomes were obtained from adjacent normal tissues. The transfer of circ_0067557 was tracked using fluorescence labeling and endocytosis assays. Expression of circ_0067557, BHLHE40, OTUB2, and EMT-related markers was assessed by qRT-PCR, Western blotting, and immunofluorescence. Gain- and loss-of-function models were established to determine the biological role of circ_0067557. RNA sequencing identified OTUB2 as a key downstream target. ChIP-qPCR and dual-luciferase reporter assays were performed to verify BHLHE40 binding to the OTUB2 promoter. RIP and RNA pull-down assays confirmed the interaction between circ_0067557 and BHLHE40. Mouse xenograft and tail vein metastasis models were used to evaluate the oncogenic effects of circ_0067557 in vivo.

**Results:**

CAF-exos successfully delivered circ_0067557 into CRC cells, significantly enhancing their proliferative, migratory, invasive, and EMT capabilities. Mechanistically, circ_0067557 interacted with and recruited BHLHE40, thereby increasing its binding to the OTUB2 promoter and promoting OTUB2 transcription. In vivo experiments further confirmed that circ_0067557 markedly enhanced tumor growth and distant metastasis through the BHLHE40/OTUB2 signaling axis.

**Conclusion:**

CAF-exo circ_0067557 promotes EMT, invasion, metastasis, and tumor progression in CRC by recruiting BHLHE40 and activating OTUB2 transcription. These findings reveal a novel mechanism by which the tumor microenvironment (TME) regulates cancer cell metastasis.

**Supplementary Information:**

The online version contains supplementary material available at 10.1186/s13062-026-00805-4.

## Graphical Abstract

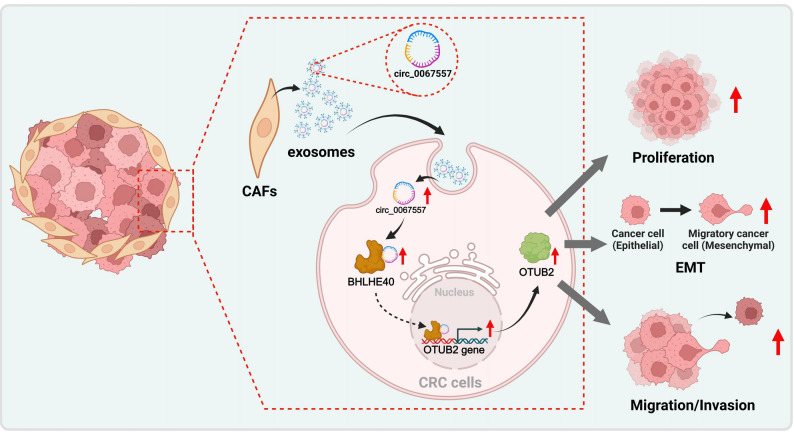

## Introduction

Colorectal cancer (CRC) is one of the most common gastrointestinal malignancies worldwide and remains a major public health challenge because of its high incidence and mortality [1, 2]. Although advances in surgical techniques and multimodal therapies have improved patient management, the five-year survival rate for advanced CRC, especially in patients with distant metastases, remains poor [2]. Metastasis is the principal cause of unfavorable prognosis in CRC [3, 4], and epithelial–mesenchymal transition (EMT) is a key biological program that drives this process [5]. During EMT, epithelial cells lose epithelial characteristics and acquire mesenchymal traits, leading to enhanced migratory and invasive potential, increased stemness, and greater therapeutic resistance [6]. Therefore, elucidating the key mechanisms underlying CRC-induced EMT is crucial for understanding the metastatic process and developing effective therapeutic interventions.

The tumor microenvironment (TME) has emerged as a critical regulator of tumor initiation and progression [7–9]. Among its stromal components, cancer-associated fibroblasts (CAFs) are particularly important because of their functional heterogeneity and dynamic interactions with cancer cells. CAFs modulate tumor behavior by secreting a wide range of cytokines and extracellular vesicles [10–12]. Among these vesicles, exosomes serve as key mediators of intercellular communication between CAFs and malignant cells by transferring diverse regulatory cargoes, including mRNAs, microRNAs, proteins, and circular RNAs (circRNAs) [13]. Increasing evidence has shown that CAF-derived exosomes (CAF-exos) promote malignant phenotypes such as proliferation, migration, immune evasion, and EMT, thereby accelerating tumor progression [14]. However, the mechanistic role of circRNAs in regulating EMT through CAF-exos in CRC remains largely unexplored. In particular, whether these circRNAs participate in transcriptional regulation and drive downstream oncogenic pathways has yet to be systematically clarified [15].

Circular RNAs (circRNAs) are a unique class of non-coding RNAs generated through back-splicing, which produces a covalently closed-loop structure and confers high stability by preventing exonuclease-mediated degradation [16, 17]. Increasing evidence has shown that circRNAs not only function as competitive endogenous RNAs by sponging microRNAs, but also regulate transcription and translation through interactions with proteins, thereby playing important roles in tumorigenesis and metastasis [18, 19]. Notably, circRNAs are highly enriched in exosomes and can exert regulatory functions across cells via extracellular delivery [20]. Some studies have suggested that certain circRNAs can form complexes with specific transcription factors, enhancing their transcriptional activity [21]. To elucidate the specific molecular mechanisms by which CAF-derived exosomes regulate malignant phenotypes in CRC, we performed transcriptome sequencing analysis on HCT116 cells treated with NF-exos and CAF-exos in our preliminary work. Among the 204 differentially expressed genes (DEGs) identified, the deubiquitinating enzyme OTUB2 showed the most significant upregulation and was closely associated with EMT pathways [22, 23]. These findings suggest that OTUB2 may play a pivotal role in CAF-induced metastasis, although its upstream transcriptional regulatory mechanism remains unclear.

To identify the key transcription factor regulating OTUB2, we used a stepwise screening strategy rather than selecting candidates arbitrarily. First, bioinformatics analysis was performed to predict transcription factors with potential binding sites in the OTUB2 promoter region. We then prioritized candidates that are highly expressed in CRC and have established roles in promoting EMT. Among the predicted factors, BHLHE40 emerged as the most promising candidate. BHLHE40 is a basic helix–loop–helix transcription factor that promotes cell migration in multiple tumor types and has been identified as a key regulator of EMT and liver metastasis in CRC [24]. Previous studies have shown that BHLHE40 is upregulated in colorectal tumors and facilitates colon cancer progression by transcriptionally activating downstream targets such as INHBA, ADAM19, and KLF7 [25, 26]. Meanwhile, OTUB2, a member of the deubiquitinating enzyme family involved in signal transduction and cell cycle regulation, has recently attracted increasing attention for its oncogenic role [22, 23]. Our preliminary bioinformatics analysis further identified putative BHLHE40 binding sites within the OTUB2 promoter. Given the critical role of BHLHE40 in CRC and its predicted binding affinity for the OTUB2 promoter, we selected BHLHE40 for further investigation. Whether circRNAs regulate OTUB2 expression through interaction with BHLHE40 has not yet been systematically explored, which forms an important basis for the novelty of the present study.

This study aimed to clarify the role of CAF-derived exosomal circ_0067557 in promoting the metastatic progression of CRC and to elucidate its underlying mechanism. Specifically, we investigated whether circ_0067557 interacts with the transcription factor BHLHE40, enhances its binding to the OTUB2 promoter, and thereby upregulates OTUB2 transcription, ultimately driving EMT and increasing the invasive capacity of CRC cells. Our findings reveal a novel mechanism by which CAFs regulate tumor cell transcriptional activity through exosome-mediated circRNA transfer, thereby expanding current understanding of how the TME promotes cancer metastasis. As an important mediator of communication between CAFs and CRC cells, circ_0067557 may serve as a potential biomarker for metastatic risk and a promising therapeutic target in CRC. These findings provide new insights into the molecular basis of CRC metastasis and offer potential avenues for precision intervention and clinical translation.

## Materials and methods

### Cell culture and grouping

Human CRC cell lines HCT116 (CCL-247), LOVO (CCL-229), and SW480 (CCL-228) were obtained from the American Type Culture Collection (ATCC, USA). HCT116 cells were cultured in Dulbecco’s modified Eagle’s medium (DMEM; 11965092, Gibco, USA), LOVO cells were maintained in F-12 K medium (N3520, Sigma-Aldrich, USA), and SW480 cells were grown in Leibovitz’s L-15 medium (L1518, Sigma-Aldrich, USA). All media were supplemented with 10% fetal bovine serum (FBS; F8687, Sigma-Aldrich, USA). The normal human colonic epithelial cell line NCM460 (HNC001, INCELL, USA) was cultured in M3:Base medium (M300A, INCELL, USA) supplemented with 10% FBS and 1% penicillin–streptomycin solution (P4333, Sigma-Aldrich, USA). All cells were maintained at 37 °C in a humidified incubator containing 5% CO_2_.

HCT116 cells were assigned to the following experimental groups: (1) normal fibroblast (NF)-derived exosomes (NF-exos) group, CAF-exos group, and CAF-exos + si-CircRNA group; (2) sh-NC + oe-NC, sh-BHLHE40 + oe-NC, and sh-BHLHE40 + oe-OTUB2.

The empty vector pcDNA3.1 and the OTUB2 overexpression plasmid were purchased from HanBio Biotechnology (Shanghai, China). Small interfering RNA targeting circ0067557 (si-CircRNA), its negative control (si-NC), shRNA targeting BHLHE40 (sh-BHLHE40), and its corresponding negative control (sh-NC) were obtained from GenePharma (Shanghai, China). The sequences of si-CircRNA were 5′-GUGUCUGCAAGCAAGUAUU-3′ and 5′-UUCACAGACGUUCGUUCAUA-3′, and the sh-BHLHE40 sequence was 5′-CATGTGAAAGCACTAACAAAC-3′. Both si-NC and sh-NC consisted of scrambled non-targeting sequences without homology to any known human gene.

For transfection, HCT116 cells were seeded into six-well plates at a density of 5 × 10^5^ cells/mL. Upon reaching approximately 80% confluence, cells were transfected with plasmids (pcDNA3.1-OTUB2 or sh-BHLHE40) or siRNAs (si-CircRNA or si-NC) using Lipofectamine 3000 (Invitrogen, USA). Cells were incubated for an additional 48 h after transfection before being subjected to subsequent experiments [27].

### Isolation and culture of fibroblasts

Fresh colorectal tumor tissues and matched adjacent normal colonic tissues were collected from 15 patients with CRC undergoing surgical resection. Adjacent normal tissues were obtained from sites located more than 5 cm away from the tumor margin and showed no histological evidence of invasion. Written informed consent was obtained from all patients, and the study protocol was approved by the institutional ethics committee. Tissue samples were immediately immersed in sterile PBS containing antibiotics, transported at 4 °C, and processed within 2 h after collection.

Tumor and matched normal tissues were washed three times with sterile PBS, minced into small pieces, and digested for 40 min in a mixed enzyme solution containing type I collagenase (SCR103), neutral protease (C0773), and hyaluronidase (H1115000) (all from Sigma-Aldrich, USA). After digestion, the cell suspensions were centrifuged at 500 rpm for 5 min, resuspended in culture medium supplemented with 12% FBS, and seeded into culture dishes. The medium was replaced on day 3 to remove tissue debris and non-adherent cells. Differential adhesion was then applied to deplete epithelial cells and enrich CAFs and NFs.

Primary CAFs and NFs were seeded onto coverslips in six-well plates. When cells reached 70%–80% confluence, they were washed with PBS and observed under an inverted microscope (CKX53, Olympus, Japan). Cell morphology was recorded using the integrated digital imaging system.

### qRT-PCR

Total RNA was extracted using TRIzol reagent (R0016, Beyotime, China), and RNA concentration and purity were assessed with a NanoDrop 2000 spectrophotometer (Thermo Fisher Scientific, USA). cDNA was synthesized using the PrimeScript RT Reagent Kit (RR047A, Takara, Japan) in a 10 µL reaction system, incubated at 37 °C for 15 min and subsequently heated to 85 °C for 5 s to terminate the reaction. Quantitative PCR was carried out using TB Green Premix Ex Taq II (Takara, Japan) on a QuantStudio 5 Real-Time PCR System (Applied Biosystems, USA). Relative gene expression was quantified by the 2^ΔΔCt^ method, with GAPDH as the internal control. Target genes included α-SMA, FAP, FSP-1, Vimentin, circ_0067557, BHLHE40, and OTUB2. Each sample group was analyzed in triplicate. Primer sequences are listed in Table S1.

### Western blot (WB)

Cells were lysed in RIPA buffer (P0013B, Beyotime, China) containing a protease inhibitor cocktail (539470, Sigma-Aldrich, USA). Protein concentrations were determined using the BCA assay (23227, Thermo Fisher Scientific, USA). Equal protein amounts (30 µg) were separated on 10% SDS-PAGE gels and transferred to PVDF membranes (IPVH00010, Millipore, USA). After blocking with 5% non-fat milk, membranes were incubated with primary antibodies against BHLHE40 (ab259837, 1:1000), α-SMA (ab7817, 1:1000), FAP (ab314456, 1:1000), FSP-1 (ab316111, 1:1000), E-cadherin (ab231303, 1:1000), N-cadherin (ab76011, 1:5000), TSG101 (ab125011, 1:1000), GAPDH (ab8245, 1:1000), OTUB2 (SAB4503136), Vimentin (V2258), CD81 (ab79559), ZO-1 (1: 1000; Abcam, ab216880), Snail (1: 1000; Abcam, ab216347), ZEB1 (1: 1000; Abcam, ab203829), and CD63 (ab134045, 1:1000). GAPDH served as the loading control. Membranes were incubated with HRP-conjugated secondary antibodies (F9137 or F9887, Sigma-Aldrich, USA), and signals were visualized using ECL reagents (Thermo Fisher Scientific, USA) on a ChemiDoc MP system (Bio-Rad, USA). Band intensities were quantified using ImageJ software (NIH, USA).

### Immunofluorescence staining

CAF and NF cells were seeded onto coverslips. When the confluency reached approximately 60–70%, the cells were fixed with 4% paraformaldehyde (PFA, 158127) for 15 min and permeabilized with 0.1% Triton X-100 (T8787), all from Sigma-Aldrich (USA) for 10 min. After blocking, the cells were incubated overnight at 4 °C with the following primary antibodies: α-SMA (ab7817, 1:1000, Abcam, UK), FAP (ab314456, 1:100, Abcam, UK), FSP-1 (ab316111, 1:1000, Abcam, UK), E-cadherin (ab231303, 1:1000, Abcam, UK), and Vimentin (V2258, 1:1000, Sigma-Aldrich, USA). The following day, cells were rinsed in PBS and incubated for 1 h at ambient temperature with fluorescence-conjugated secondary antibodies (SAB4600030 or SAB4600004, 1:500, Sigma-Aldrich, USA). For F-actin staining, cells were incubated with 200 µL of fluorescein isothiocyanate (FITC)-labeled phalloidin working solution (Actin-Tracker Green-488; Cat. No. C2201S, Beyotime, China) for 30 min at room temperature. Nuclear staining was achieved using DAPI (P0131, Beyotime, China). Fluorescent images were captured with a confocal microscope (Zeiss LSM800, Germany) and processed using Zen Blue software (Zeiss, Germany).

### Isolation and extraction of exosomes

CAF and NF cells were washed twice with PBS (C0221A, Beyotime, China) at 70–80% confluence and cultured in DMEM supplemented with exosome-depleted FBS (EXO-FBS-50 A-1, System Biosciences, USA) for 48 h. After a further 48 h incubation, conditioned media from CAFs and NFs were collected. The supernatants were sequentially centrifuged (300 × g, 10 min; 2,000 × g, 20 min; 10,000 × g, 30 min) to remove cells, debris, and large vesicles. Exosomes were then isolated by ultracentrifugation (100,000 × g, 90 min) using an Optima XPN-100 ultracentrifuge with an SW41Ti rotor. The pellets were resuspended in PBS and washed by a second ultracentrifugation at 100,000 × g. Final exosome preparations were resuspended in 100 µL PBS and designated as CAF-exos or NF-exos. All procedures were performed at 4 °C.

### Transmission electron microscopy (TEM) of exosome morphology

A 10 µL aliquot of exosome suspension was applied to a carbon-coated copper grid (Beijing Zhongjingkeyi Technology Co., China) and allowed to adsorb for 10 min. The grid was then negatively stained with 2% phosphotungstic acid (pH 7.0; P4006, Sigma-Aldrich, USA) for 30 s and air-dried using filter paper. Exosome morphology was examined with a transmission electron microscope (JEM-1400 Plus, JEOL, Japan) at an accelerating voltage of 120 kV, and images were captured to verify the characteristic cup-shaped vesicular structure.

### Nanoparticle tracking analysis (NTA) for particle size distribution

Exosome size distribution and concentration were determined using a ZetaView PMX 110 instrument (Particle Metrix, Germany) and analyzed with ZetaView software (version 8.05.14). Samples were diluted in sterile PBS to a final concentration of 1 × 10^8^ to 1 × 10^9^ particles/mL. For each sample, particle movement was recorded in 11 independent fields, and the mean particle size and distribution profile were automatically generated by the system.

### Fluorescence labeling of exosomes

CAF-exos or NF-exos were resuspended in 500 µL Diluent C and incubated with 2 µL PKH67 dye (PKH67GL, Sigma-Aldrich, USA) at 37 °C in the dark for 5 min. The labeling reaction was quenched by adding an equal volume of 0.5% BSA (A2153, Sigma-Aldrich, USA). Excess dye was removed by ultracentrifugation (100,000 × g, 90 min). The pellets were washed twice and resuspended in 100 µL PBS. Unlabeled exosomes were used as negative controls.

### Endocytosis assays of exosomes

HCT116, SW480, and LoVo cells were seeded onto coverslips in 24-well plates (Nest, China) and cultured to 60–70% confluence. The medium was replaced with DMEM containing exosome-depleted FBS, and PKH67-labeled CAF-exos or NF-exos were added at a final concentration of 1 × 10^9^ particles/mL. PBS-treated cells served as negative controls. Cells were incubated at 37 °C for 2, 6, 12, or 24 h, washed three times with PBS at each time point, and fixed with 4% PFA for 15 min.

### Confocal laser scanning microscopy

After fixation, cells were stained with DAPI (1 µg/mL) for 10 min, washed with PBS, and mounted for imaging. Fluorescence images were acquired using a Zeiss LSM800 confocal microscope (Germany). PKH67-labeled exosomes were detected under 488 nm excitation, and nuclei were visualized under 405 nm excitation. Image acquisition and analysis were performed using Zen Blue software (Zeiss, Germany). All images were captured under identical exposure settings to ensure comparability.

### Assessment of exosome uptake efficiency

Exosome uptake efficiency in the time-course experiment was evaluated by calculating the percentage of PKH67-positive cells in each image. For each group, five random fields were analyzed, and at least 300 cells were counted in total. Cells showing green fluorescent puncta in the cytoplasm were considered positive. Quantification was performed using ImageJ software (NIH, USA) with the Cell Counter plugin. Data were expressed as the percentage of PKH67-positive cells. All experiments were performed in triplicate.

### CCK-8

HCT116 cells were seeded in 96-well plates (Corning, USA) at 5 × 10^3^ cells per well, with five replicate wells per group. After cell attachment, the culture medium was replaced with antibiotic-free medium, and cells were subjected to the indicated treatments. At 0, 24, 48, and 72 h, 10 µL Cell Counting Kit-8 (CCK-8) reagent was added to each well and incubated for 2 h. Absorbance was then measured at 450 nm using a Synergy H1 microplate reader (BioTek, USA).

### Colony formation assay

HCT116 cells were seeded into 6-well plates (Nest, China) at 500 cells per well and cultured for 10–14 days, with medium changes every three days. When visible colonies developed, cultures were washed with PBS and fixed in 4% PFA for 15 min, and stained with 0.1% crystal violet (C8470, Solarbio, China) for 30 min. After excess dye was removed by rinsing with running water, the plates were air-dried and photographed. Colony numbers were quantified using ImageJ software (NIH, USA). Three replicate wells were included in each group.

### Transwell migration and invasion assays

Cell migration and invasion were assessed using Transwell inserts with 8 μm pores (Corning, USA). For migration, 2 × 10^4^ treated HCT116 cells in serum-free DMEM were added to the upper chamber, while medium containing 10% FBS was placed in the lower chamber. For invasion, inserts were precoated with Matrigel (50 µL; 354230, Corning) for 2 h at 37 °C, and 2 × 10^5^ cells were seeded. After 24 h, cells remaining on the upper surface were removed, and the membranes were washed with PBS, fixed in 4% PFA, and stained with 0.1% crystal violet. Migrated or invaded cells were counted in five random fields per well.

### Wound healing assay

HCT116 cells were grown in 6-well plates until the monolayer exceeded 90% confluence. A straight wound was generated using a sterile 200 µL pipette tip, and cell debris was removed by gentle washing. Serum-free DMEM was then added. Phase-contrast images were taken at 0 and 24 h using a Nikon Eclipse TS2 microscope (Japan). Wound width was quantified in ImageJ, and migration was expressed as: Closure rate = [(initial width − 24 h width) / initial width] × 100% [28].

### RNA sequencing and differential expression analysis

Total RNA was extracted from HCT116 cells treated with NF-exos or CAF-exos for 24 h (*n* = 3 per group) using TRIzol reagent. RNA quality was evaluated with an Agilent 2100 Bioanalyzer (Agilent, USA), and samples with OD260/280 ratios of 1.8–2.0 and RNA integrity numbers ≥ 8.0 were used for library preparation. Sequencing libraries were constructed using the NEBNext Ultra RNA Library Prep Kit (New England Biolabs, USA) and sequenced on the Illumina NovaSeq 6000 platform to generate 150-bp paired-end reads. Raw FASTQ files were quality-checked with FastQC (v0.11.9), aligned to the human reference genome (hg38) using HISAT2 (v2.2.1), and quantified with FeatureCounts (v2.0.3). DEGs were identified using DESeq2 (v1.38.3), with the thresholds set at |log2 fold change| > 1 and adjusted *p* < 0.05.

Functional enrichment analysis was conducted with ClusterProfiler (v4.6.0) in R. GO and KEGG terms were evaluated using Benjamini–Hochberg correction (adjusted *p* < 0.05). GO categories included biological process, cellular component, and molecular function, and KEGG annotations were obtained from the KEGG database (https://www.kegg.jp/).

### Chromatin immunoprecipitation followed by qPCR (ChIP-qPCR)

ChIP assays were performed using the Millipore ChIP Assay Kit (17–295, Millipore, USA). HCT116 cells were fixed in 1% formaldehyde (47673, Sigma-Aldrich, USA)for 10 min, and the reaction was then quenched. Chromatin was isolated and fragmented to 200–500 bp by sonication using a Bioruptor Pico system (Diagenode, Belgium). Equal amounts of chromatin were incubated overnight with an anti-BHLHE40 antibody (ab230924, Abcam, UK) or normal IgG as the negative control (Cell Signaling Technology, USA). Immune complexes were isolated with Protein A/G magnetic beads, eluted, de-crosslinked, and purified for downstream DNA analysis. Quantitative PCR was conducted to amplify the OTUB2 promoter-specific sequence. ChIP signal intensity was normalized to input DNA and presented as %IP/Input.

### Dual-luciferase reporter assay

To investigate whether BHLHE40 regulates OTUB2 expression by binding to its promoter, the OTUB2 promoter region (~ 2 kb) was amplified using a forward primer containing an NheI restriction site (5′-GCTAGCCTGTTACGCATGCACTGAGATT-3′) and a reverse primer containing an XbaI restriction site (5′-TCTAGACCCCCACGATCACTGACCATAG-3′) (Sangon Biotech, Shanghai, China). The amplified fragment was inserted into the NheI and XbaI sites of the pGL3-basic vector (GenBank accession no. U47295; Promega, USA) to generate the wild-type OTUB2 promoter reporter (WT). A mutant reporter (MUT) lacking the predicted BHLHE40-binding sequence at nucleotides 2925–2934 was also constructed. For promoter activity analysis, WT or MUT reporter plasmids were co-transfected with the Renilla luciferase control plasmid pRL-TK into HCT116 cells using Lipofectamine 3000 (Invitrogen, USA). To assess the effect of circ_0067557 on OTUB2 transcription, HCT116 cells were assigned to the NF-exos, CAF-exos, and CAF-exos + si-circ_0067557 groups. Cells in each group were co-transfected with the WT reporter and pRL-TK under the indicated treatment conditions. After 48 h, luciferase activity was measured using the Dual-Luciferase Reporter Assay System (Promega, USA) on a SpectraMax i3x microplate reader (Molecular Devices, USA). Relative promoter activity was expressed as the ratio of Firefly to Renilla luciferase activity. Each experiment was performed with three biological replicates [29, 30].

### RNA pull-down assay

To confirm the potential interaction between circ_0067557 and BHLHE40, streptavidin-coupled magnetic beads (100 µL) were pre-treated by incubating with 0.5 g/L yeast tRNA and 1% RNase-free BSA. The beads were then incubated with 100 pmol of biotinylated circ_0067557 or a control probe (25 µL) for 3 h at 4 °C. Biotinylated circ_0067557 was synthesized by Geneseed (Guangzhou, China). The probe-bound beads were subsequently incubated with 750 µL of cell lysate for 3 h at 4 °C, followed by centrifugation. An aliquot of the supernatant (50 µL) was retained as the input control. After binding, the RNA-protein complexes were eluted, and BHLHE40 was detected by WB [31].

### RNA immunoprecipitation (RIP) assay

RIP assays were performed using the Magna RIP Kit (17–700, Millipore, USA). HCT116 cells were lysed in RIP buffer and incubated overnight with anti-BHLHE40 antibody (Abcam, UK) or normal IgG as a negative control, together with Protein A/G magnetic beads. After washing, the immunoprecipitated RNA–protein complexes were lysed, and the associated RNA was purified. Enrichment of circ_0067557 was quantified by qRT–PCR and normalized to input RNA. All assays were performed in triplicate.

### Molecular docking

The predicted structure of BHLHE40 (UniProt ID: O14503) was obtained from the AlphaFold database (AlphaFold ID: AF-O14503-F1), and the crystal structure of OTUB2 was retrieved from the Protein Data Bank (PDB ID: 4FJV). Protonation states were adjusted to pH 7.4, and all structures were converted into three-dimensional format using Open Babel. circ_0067557, BHLHE40, and OTUB2 were prepared with AutoDock Tools (version 1.5.7). Docking grids were generated using AutoGrid, and docking simulations were carried out with AutoDock Vina (version 1.2.0). The optimal docking conformation was selected based on binding affinity for subsequent interaction analysis. Molecular interactions were visualized using PyMOL (version 3.1).

### Colocalization of circ_0067557 and BHLHE40

To examine the subcellular colocalization of circ_0067557 and BHLHE40, RNA fluorescence in situ hybridization (FISH) combined with immunofluorescence staining was performed in HCT116 cells. A Cy3-labeled probe targeting circ_0067557 was designed and synthesized by Sangon Biotech (Shanghai, China). Detection of circ_0067557 was carried out using a FISH kit (F32202/50, GenePharma, Shanghai, China). Briefly, cells were fixed with ethanol for 15 min, permeabilized with Triton X-100 for 15 min, and hybridized overnight at 37 °C with the denatured probe mixture provided by the kit. After hybridization, cells were incubated overnight at 4 °C with an anti-BHLHE40 antibody (PA1-16546, 1:200, Thermo Fisher Scientific, USA). On the following day, cells were washed with PBS and incubated with a fluorescence-conjugated secondary antibody (SAB4600030, 1:500, Sigma-Aldrich, USA) for 1 h at room temperature. Nuclei were counterstained with DAPI (P0131, Beyotime, China) for 20 min. Fluorescence images were acquired using a Zeiss LSM800 confocal microscope (Germany) [32].

### Model construction and grouping

HCT116 cells (5 × 10^6^ in 100 µL PBS) were injected subcutaneously into the axillary region of 4-week-old male BALB/c nude mice (20 ± 2 g, specific pathogen-free grade; Shanghai Slack Laboratory Animal Co., Ltd., China). When tumor volumes reached 50–100 mm^3^, mice were randomly assigned to different treatment groups. Tumor length and width were measured weekly, and tumor volume was calculated as V = (L × W^2^)/2. After 4 weeks, mice were euthanized and tumors were excised and weighed. All animal experiments were approved by the Animal Ethics Committee of Harbin Medical University Cancer Hospital.

For the metastatic model, HCT116 cells (1 × 10^6^ cells per mouse) were administered via tail vein injection into 6-week-old male nude mice. After six weeks, mice were sacrificed, and lungs and livers were harvested and fixed in 10% neutral-buffered formalin for 24 h. Metastatic foci were evaluated macroscopically and examined through histological sectioning. Two independent pathologists assessed and quantified metastatic lesions in a blinded manner.

Mice were assigned to four groups: NF-exos group (injected with 10 µg, 100 µL NF-exos), CAF-exos group (10 µg, 100 µL CAF-exos), CAF-exos + siCircRNA group (10 µg, 100 µL CAF-exos with siCircRNA), and CAF-exos + siCircRNA + oe-OTUB2 group (10 µg, 100 µL CAF-exos with siCircRNA and oe-OTUB2). Each group included six mice [33, 34].

### Immunohistochemistry

Paraffin-embedded tissues were cut into 4 μm sections. Antigen retrieval was achieved by heating sections in citrate buffer (pH 6.0, Beyotime, China) for 15 min. Endogenous peroxidase activity was quenched with 3% H_2_O_2_ for 10 min at ambient temperature. After a 5% BSA block (30 min), sections were incubated overnight at 4 °C with primary antibodies against Ki67 (ab15580, 1:200), CD31 (ab281583, 1:4000), E-cadherin (ab231303, 1:1000), N-cadherin (ab76011, 1:1000), and Vimentin (V2258, 1:1000). The next day, slides were washed and incubated with HRP-conjugated secondary antibodies (F9137 or F9887) for 30 min, followed by DAB visualization and hematoxylin counterstaining. After dehydration and mounting, images were acquired using an Olympus BX53 microscope (Japan).

### Statistical analysis

Experiments were performed in triplicate, and data were reported as mean ± SD. Two-tailed t-tests were used for comparisons between two groups, while one-way ANOVA followed by Tukey’s test was applied for multiple-group analysis. Statistical analysis was conducted using GraphPad Prism 9.0 (GraphPad Software, USA). A *p*-value < 0.05 was considered statistically significant.

## Results

### CAF-exo circ_0067557 can be transferred to CRC cells

In this study, CAFs and NFs were obtained from CRC tissues and matched adjacent normal mucosa. Cell morphology was observed under a microscope (Figure S1A). qRT-PCR, WB, and immunofluorescence analyses showed markedly elevated levels of α-SMA, FAP, FSP-1, and Vimentin in CAFs, whereas NFs displayed only minimal expression of these markers (Figure S1B-D).

**Fig. 1:**
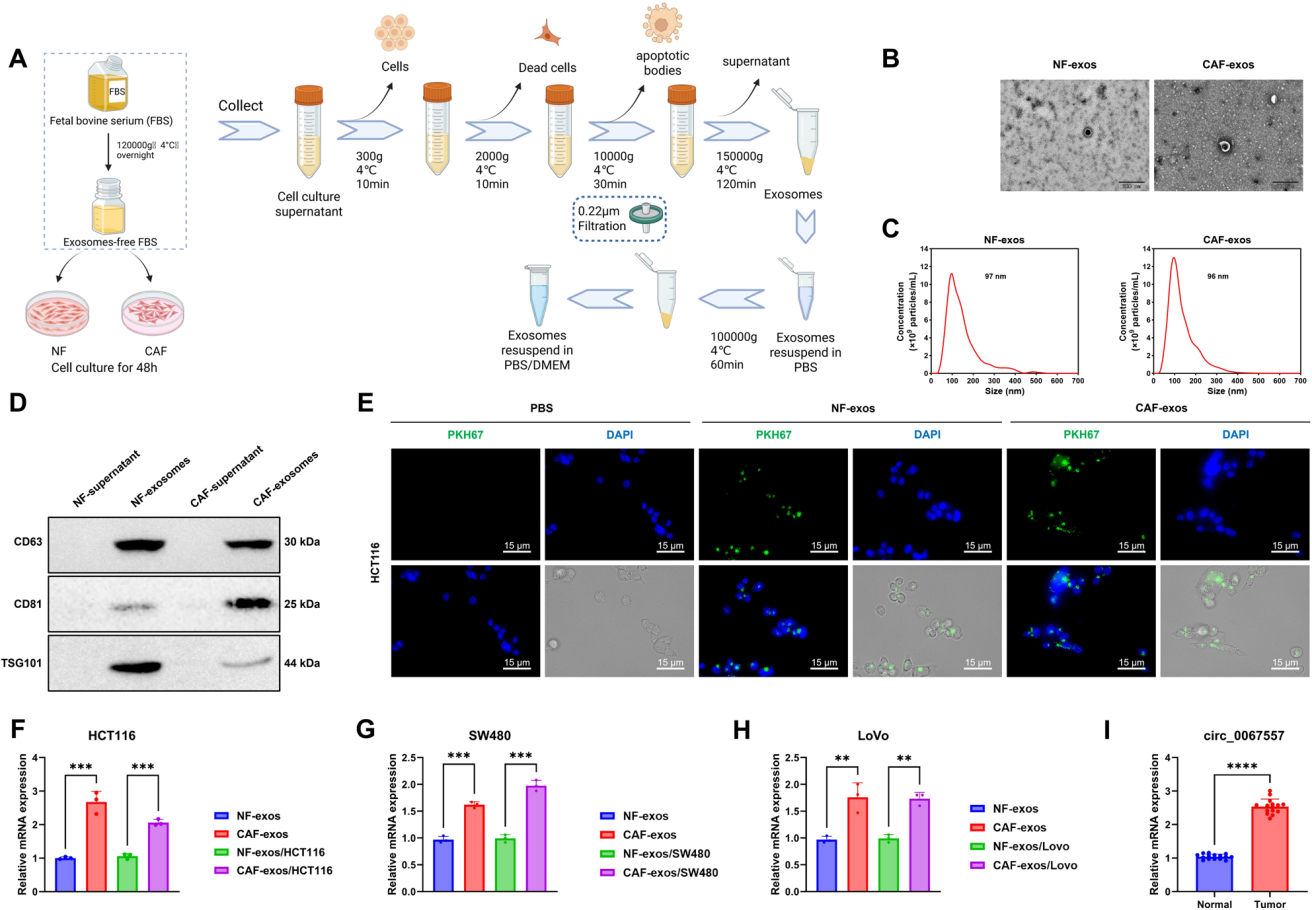
Isolation and characterization of CAF-exos and verification of circ_0067557 transfer to CRC cells. Note: (**A**) Exosomes were isolated from CAF-CM or NF-CM using differential ultracentrifugation; (**B**) TEM imaging of CAF-exos morphology. Scale bar: 200 μm; (**C**) NTA of CAF-exos particle size distribution; (**D**) WB analysis of exosomal markers CD63, CD81, and TSG101 in CAF-exos and NF-exos; (**E**) LSCM visualization of PKH67-labeled CAF-exos and NF-exos internalized by HCT116 cells. Scale bar: 25 μm; (**F**) qRT-PCR analysis of circ_0067557 expression in CAF-exos, NF-exos, and in HCT116 cells treated with each; (**G**-**H**) Expression of circ_0067557 in SW480 and LoVo cells following similar treatments; (**I**) qRT-PCR detection of circ_0067557 expression in CRC tissues (Tumor) and matched adjacent normal mucosa (Normal) (*N* = 15). All cell-based experiments were performed in triplicate. *p < 0.05, **p < 0.01, ***p < 0.001 (between groups)

Exosomes were subsequently isolated from CAF-CM and NF-CM using differential ultracentrifugation (Fig. 1A). TEM revealed the typical cup-shaped morphology of the isolated vesicles (Fig. 1B), while NTA showed that most particles ranged from 50 to 100 nm in diameter, consistent with the known size distribution of exosomes (Fig. 1C). WB analysis further confirmed the presence of the exosomal marker proteins CD63, CD81, and TSG101 (Fig. 1D). These results confirmed the successful isolation of CAF-exos and NF-exos.

To determine whether exosomes released by CAFs and NFs could be internalized by CRC cells, PKH67-labeled NF-exos and CAF-exos were incubated with CRC cells. Confocal microscopy showed clear green fluorescence in HCT116 cells treated with either NF-exos or CAF-exos, whereas no fluorescence signal was detected in PBS-treated control cells (Fig. 1E). Similar uptake patterns were observed in SW480 and LOVO cells (Figure S2A), indicating efficient internalization of fibroblast-derived exosomes by CRC cells. Time-course analysis further showed that the proportion of PKH67-positive cells gradually increased in all three CRC cell lines, with more than 90% of cells showing positive fluorescence after 24 h. No significant difference in uptake efficiency was observed between CAF-exos and NF-exos in any of the tested CRC cell lines (Figure S2B).

Our previous transcriptome sequencing data showed that circ_0067557 was markedly enriched in CAF-derived exosomes, suggesting its potential involvement in CRC progression [27]. Based on this observation, qRT-PCR was performed to examine circ_0067557 expression in NF-exos, CAF-exos, and HCT116 cells treated with the corresponding exosomes. Four groups were analyzed, including NF-exos, CAF-exos, NF-exos/HCT116, and CAF-exos/HCT116. circ_0067557 expression was significantly higher in CAF-exos than in NF-exos. Consistently, HCT116 cells exposed to CAF-exos exhibited markedly increased circ_0067557 levels compared with cells treated with NF-exos (Fig. 1F). The same trend was also confirmed in SW480 and LOVO cells (Fig. 1G-H). In addition, qRT-PCR analysis of clinical samples showed that circ_0067557 expression was significantly elevated in CRC tissues compared with matched adjacent normal mucosa (Fig. 1I).

Collectively, these findings demonstrate that CAF-exo circ_0067557 can be transferred into CRC cells.

### CAF-exo circ_0067557 promotes EMT in CRC cells

**Fig. 2:**
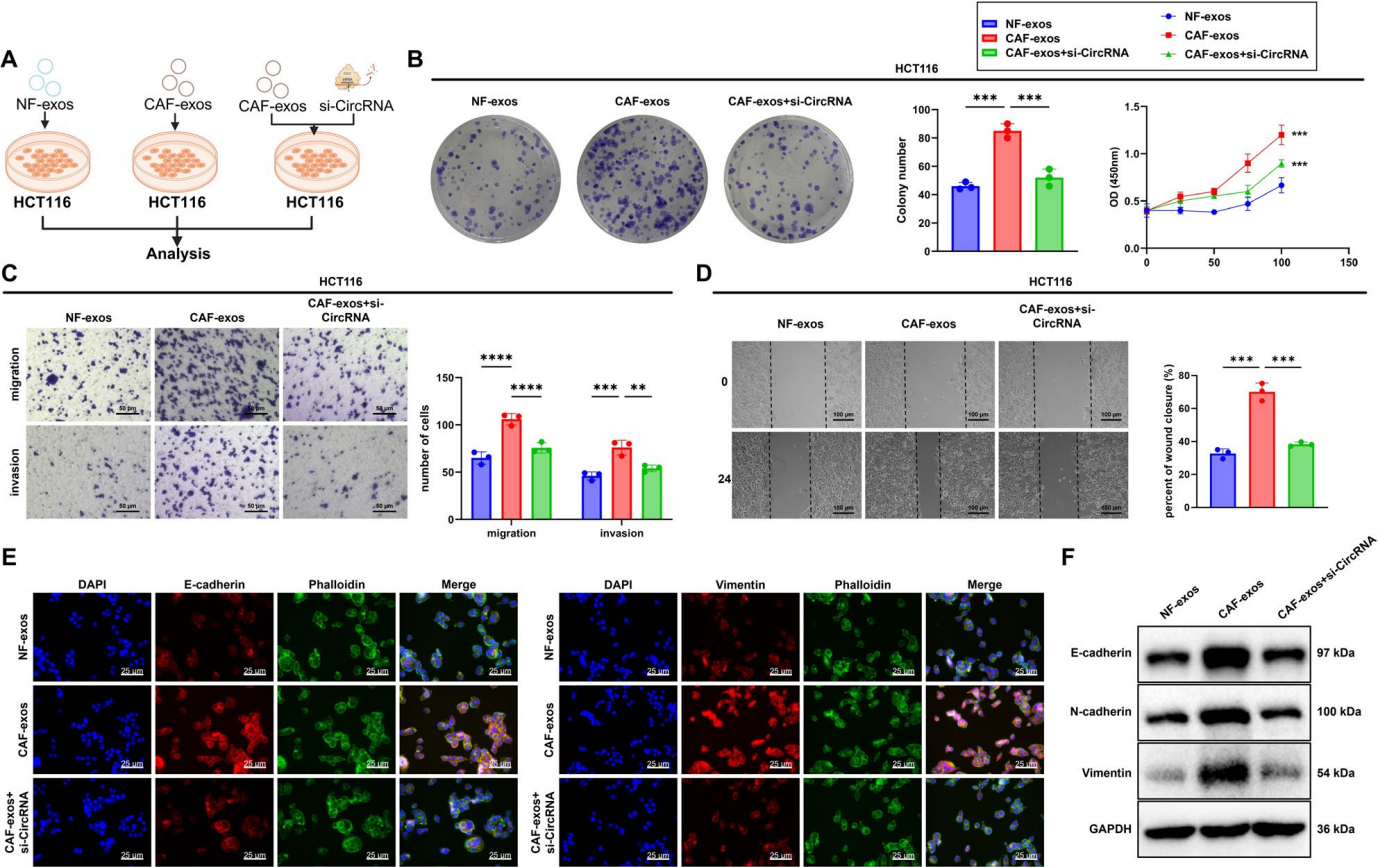
CAF-exo circ_0067557 promotes EMT progression in CRC cells. Note: (**A**) Schematic diagram of the experimental design. HCT116 cells were divided into three groups: NF-exos, CAF-exos, and CAF-exos + si-CircRNA; (**B**) Cell proliferation was assessed using colony formation and CCK-8 assays. Scale bar: 500 μm; (**C**) Transwell assays evaluated cell migration and invasion. Scale bar: 50 μm; (**D**) Wound healing assays measured cell migration. Scale bar: 100 μm; (**E**) Immunofluorescence staining assessed the expression and localization of EMT markers E-cadherin and Vimentin. Scale bar: 25 μm; (**F**) WB measured the expression of EMT-related proteins E-cadherin, N-cadherin, and Vimentin in the three groups. All experiments were performed in triplicate. *p < 0.05, **p < 0.01, ***p < 0.001 (between groups)

We further examined the role of circ_0067557 by knocking down its expression during CAF-exos treatment. Specifically, HCT116 cells were assigned to three groups: NF-exos, CAF-exos, and CAF-exos + si-CircRNA (workflow shown in Fig. 2A). Cell proliferation and clonogenic ability were assessed using CCK-8 and colony formation assays, whereas migration and invasion were evaluated by Transwell and wound healing assays. The expression of EMT-related markers, including E-cadherin, N-cadherin, and Vimentin, was analyzed by immunofluorescence and WB. Compared with the NF-exos group, the CAF-exos group exhibited enhanced proliferation, colony formation, migration, invasion, and significantly upregulated expression of EMT markers. In contrast, compared with the CAF-exos group, silencing circ_0067557 in the presence of CAF-exos markedly attenuated these malignant phenotypes, as evidenced by reduced proliferative, clonogenic, migratory, and invasive capacities, together with decreased expression of EMT markers, compared with the CAF-exos group. Notably, no obvious difference was observed in actin filament staining among the three groups (Fig. 2B-F). These results indicate that CAF-derived exosomal circ_0067557 promotes EMT and malignant progression in CRC cells. Knockdown of circ_0067557 effectively reversed these effects without altering the intracellular filamentous architecture of HCT116 cells, highlighting a critical regulatory role of CAF-exosomal circ_0067557 in CRC progression.

### OTUB2 is a key target gene of circ_0067557

**Fig. 3:**
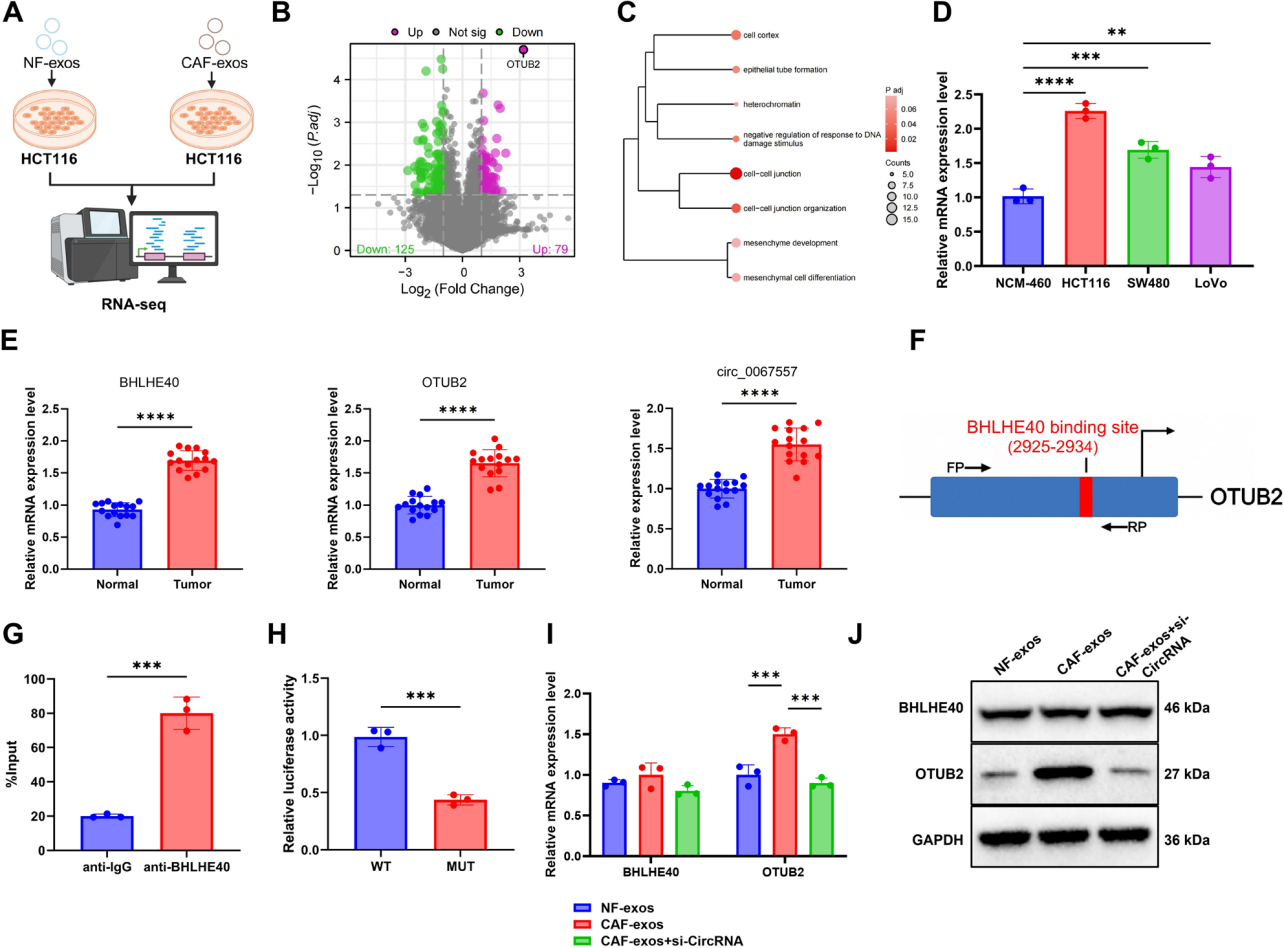
Mechanistic analysis of circ_0067557-mediated regulation of downstream gene OTUB2 expression. Note: (**A**) Schematic diagram of the RNA sequencing workflow analyzing HCT116 cells treated with NF-exos or CAF-exos; (**B**) Volcano plot showing differentially expressed genes, with OTUB2, BHLHE40, and circ_0067557 exhibiting the most significant change; (**C**) GO and KEGG enrichment analysis of differentially expressed genes; (**D**) qRT-PCR analysis of OTUB2 expression in CRC cell lines and normal colonic epithelial cells; (**E**) qRT-PCR analysis of OTUB2 expression in CRC tissues (Tumor) and matched normal mucosal tissues (Normal) (*N* = 15); (**F**) Schematic of BHLHE40 binding site within the OTUB2 promoter region. The red segment indicates the BHLHE40 binding site located at nucleotides 2925–2934; FP and RP represent forward and reverse primer locations used for subsequent ChIP-qPCR analysis; (**G**) ChIP-qPCR assay evaluating the enrichment of BHLHE40 binding to the OTUB2 promoter region; (**H**) Dual-luciferase reporter assay assessing the regulatory effect of BHLHE40 on OTUB2 promoter activity; (**I**-**J**) qRT-PCR (**I**) and WB (**J**) analyses of BHLHE40 and OTUB2 expression in HCT116 cells across NF-exos, CAF-exos, and CAF-exos + si-CircRNA groups. All cell-based experiments were performed in triplicate. *p < 0.05, **p < 0.01, ***p < 0.001 (between groups)

RNA sequencing was performed on HCT116 samples exposed to NF-exos or CAF-exos (Fig. 3A). Using a threshold of |log_2_FC| > 1 and adjusted *p <* 0.05, 204 DEGs were identified, among which OTUB2 showed the most significant change (Fig. 3B). GO and KEGG enrichment analyses indicated that these genes were mainly enriched in pathways related to EMT (Fig. 3C). We further validated the differential expression of OTUB2, BHLHE40, and circ_0067557 in CRC cell lines using qRT-PCR. Compared with the normal colonic epithelial cell line NCM460, OTUB2, BHLHE40, and circ_0067557 expression were significantly upregulated in HCT116, SW480, and LoVo cells (Fig. 3D). Consistently, OTUB2 expression was also markedly elevated in CRC tissues relative to matched adjacent normal mucosa (Fig. 3E).

Previous studies have shown that circRNAs can regulate downstream gene expression through interactions with transcription factors. Based on our preliminary bioinformatic analysis, a putative BHLHE40 binding site was identified within the OTUB2 promoter region (Fig. 3F). ChIP-qPCR analysis demonstrated significant enrichment of the OTUB2 promoter fragment in the anti-BHLHE40 immunoprecipitation group compared with the IgG control, confirming that BHLHE40 directly binds to the OTUB2 promoter (Fig. 3G). Consistently, dual-luciferase assays showed that mutation of the predicted binding site markedly reduced OTUB2 promoter activity (Fig. 3H). These data indicate that BHLHE40 functions as a transcriptional regulator of OTUB2.

To further examine whether circ_0067557 affects OTUB2 expression through BHLHE40, the expression levels of BHLHE40 and OTUB2 were measured in HCT116 cells treated with NF-exos, CAF-exos, or CAF-exos + si-circ_0067557. Compared with the NF-exos group, OTUB2 mRNA and protein levels were markedly increased in the CAF-exos group. Silencing circ_0067557 significantly attenuated this upregulation, resulting in lower OTUB2 expression than that in the CAF-exos group. In contrast, BHLHE40 expression at both the mRNA and protein levels remained unchanged among the three groups (Fig. 3I-J). These findings indicate that OTUB2 is a transcriptional target of BHLHE40 and suggest that circ_0067557 may participate in this regulatory process.

### circ_0067557 activates OTUB2 transcription to enhance EMT in CRC cells

**Fig. 4:**
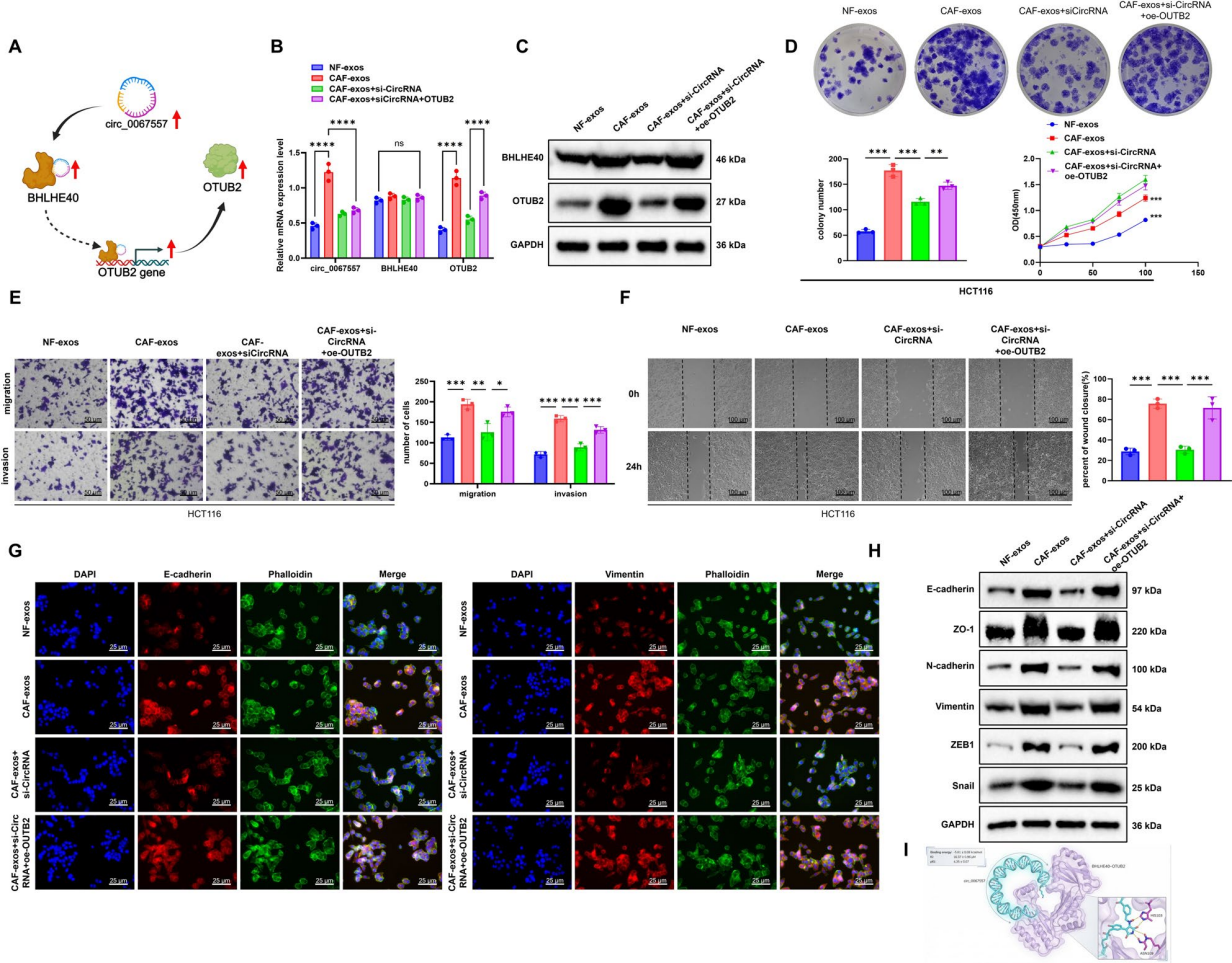
circ_0067557 activates OTUB2 transcription to enhance EMT capabilities in CRC cells. Note: (**A**) Schematic illustration of the proposed mechanism: circ_0067557 promotes OTUB2 expression by recruiting the transcription factor BHLHE40 to activate OTUB2 transcription; (**B**) qRT-PCR analysis of circ_0067557, BHLHE40, and OTUB2 mRNA expression levels in HCT116 cells; (**C**) Western Blot analysis of BHLHE40 and OTUB2 protein expression levels in HCT116 cells; (**D**) Colony formation and CCK-8 assays assessing the proliferative capacity of HCT116 cells. Scale bar: 500 μm; (**E**) Transwell assays evaluating the migration and invasion capabilities of HCT116 cells. Scale bar: 50 μm; (**F**) Wound healing assay measuring the migratory ability of HCT116 cells. Scale bar: 100 μm; (**G**) Immunofluorescence staining detecting the expression and localization of EMT-related marker proteins E-cadherin and Vimentin in HCT116 cells. Actin filaments were visualized using phalloidin. Scale bar: 25 μm; (**H**) Western Blot analysis of the expression levels of EMT-related marker proteins E-cadherin, ZO-1, N-cadherin, Vimentin, Snail, and ZEB1 in HCT116 cells; (**I**) Molecular docking analysis of the circ_0067557–BHLHE40–OTUB2 interaction. All cell-based experiments were performed in triplicate. *p < 0.05, **p < 0.01, ***p < 0.001 (between groups)

Our previous findings suggested that circ_0067557 may enhance OTUB2 expression by recruiting the transcription factor BHLHE40 to activate OTUB2 transcription (schematic diagram shown in Fig. 4A). To further validate this mechanism, HCT116 cells were divided into four groups: NF-exos, CAF-exos, CAF-exos + si-circ_0067557, and CAF-exos + si-circ_0067557 + oe-OTUB2. The expression levels of circ_0067557, BHLHE40, and OTUB2 were examined by qRT-PCR and WB. Compared with the NF-exos group, circ_0067557 and OTUB2 expression was significantly increased in the CAF-exos group. Silencing circ_0067557 markedly reduced OTUB2 expression, whereas OTUB2 overexpression restored its level in the CAF-exos + si-circ_0067557 + oe-OTUB2 group. In contrast, BHLHE40 expression remained unchanged across all groups (Fig. 4B, C). These results suggest that circ_0067557 regulates OTUB2 expression without affecting BHLHE40 abundance.

Functional assays were then performed to assess the biological consequences of this regulatory axis. CCK-8 and colony formation assays showed that CAF-exos significantly enhanced cell proliferation and clonogenicity compared with NF-exos, whereas circ_0067557 knockdown attenuated these effects. Similarly, Transwell and wound healing assays demonstrated that CAF-exos promoted the migratory and invasive capacities of HCT116 cells, while silencing circ_0067557 markedly suppressed these malignant phenotypes. Importantly, OTUB2 overexpression largely reversed the inhibitory effects caused by circ_0067557 knockdown. Consistently, immunofluorescence and WB analyses showed that CAF-exos induced marked upregulation of EMT-related markers, including N-cadherin, Vimentin, Snail, and ZEB1, together with downregulation of epithelial markers such as E-cadherin and ZO-1. These changes were significantly reversed by circ_0067557 silencing and were restored after OTUB2 overexpression. No obvious differences were observed in actin filament staining among the groups (Fig. 4D-H).

To further support the interaction among circ_0067557, BHLHE40, and OTUB2, molecular docking analysis was performed. The results showed that circ_0067557 formed hydrogen bonds with HIS103 of BHLHE40 and ASN109 of OTUB2, yielding a binding energy of 5.33 ± 0.09 kcal/mol and a binding affinity of 4.76 ± 0.03, indicative of favorable molecular interactions (Fig. 4I). Collectively, these findings demonstrate that circ_0067557 promotes EMT and malignant progression in CRC cells by enhancing OTUB2 transcription through a BHLHE40-dependent mechanism.

### circ_0067557 promotes OTUB2 expression by recruiting BHLHE40

**Fig. 5:**
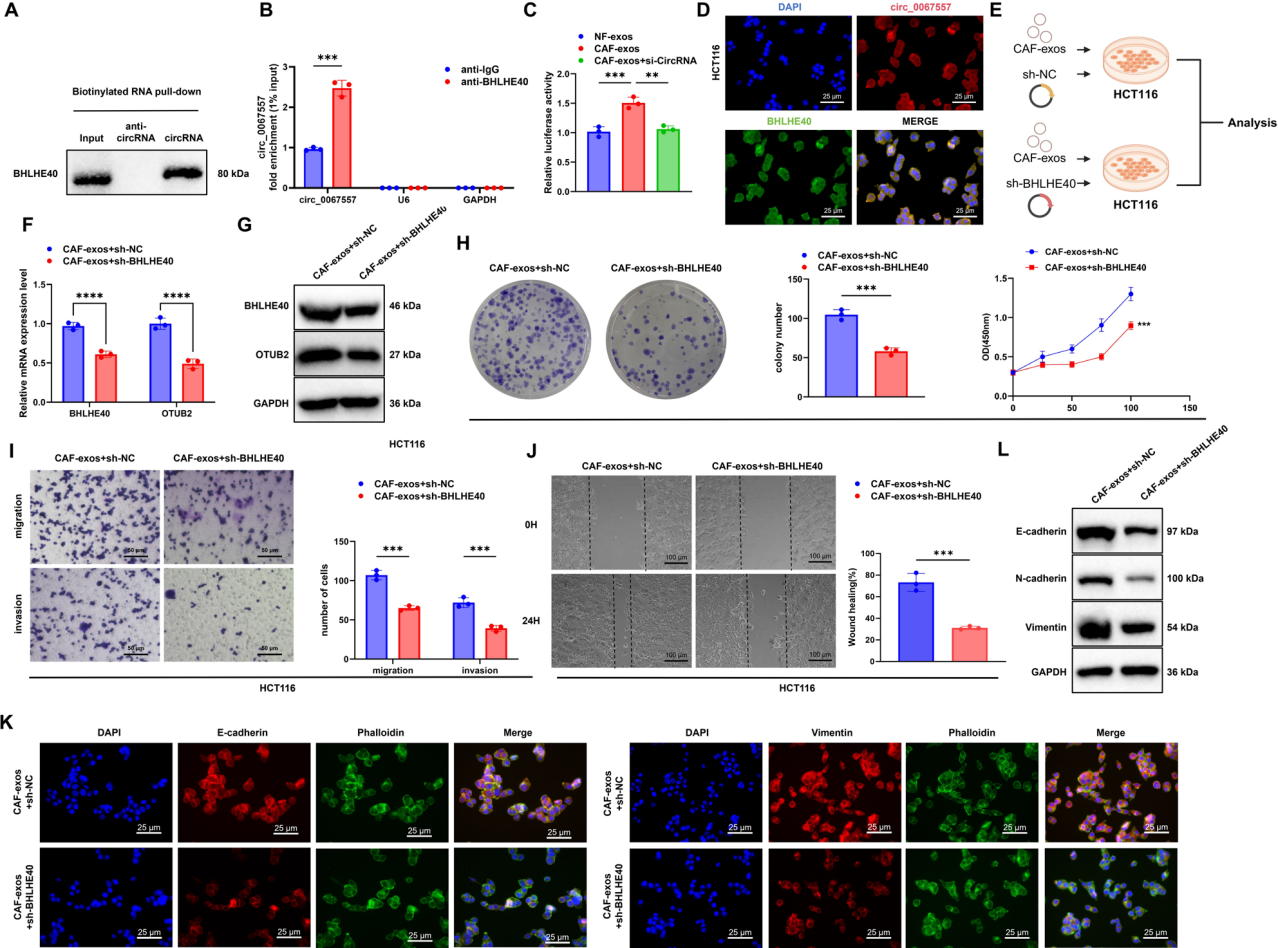
circ_0067557 promotes OTUB2 expression and enhances EMT in CRC cells by recruiting BHLHE40. Note: (**A**) RNA pull-down assay evaluating the enrichment of BHLHE40 protein by circ_0067557; (**B**) RIP assay assessing the enrichment of circ_0067557 by BHLHE40 protein; (**C**) Dual-luciferase reporter assay validating the regulatory effect of circ_0067557 on OTUB2 transcriptional activity; (**D**) Colocalization of circ_0067557 and BHLHE40 in HCT116 cells. Scale bar: 50 μm; (**E**) Experimental grouping schematic: HCT116 cells were divided into CAF-exos + sh-NC and CAF-exos + sh-BHLHE40 groups; (**F**-**G**) qRT-PCR (**F**) and WB (**G**) analyses of BHLHE40 and OTUB2 expression levels in the two groups; (**H**) Colony formation and CCK-8 assays evaluating the proliferation capacity of HCT116 cells in both groups, bar = 500 μm; (**I**) Transwell assays assessing the migration and invasion abilities of HCT116 cells, bar = 50 μm; (**J**) Wound healing assays measuring cell migration, bar = 100 μm; (**K**) Immunofluorescence staining showing the expression and localization of EMT-related markers E-cadherin and Vimentin in HCT116 cells, bar = 25 μm; (**L**) WB analysis of EMT-related markers E-cadherin, N-cadherin, and Vimentin in the two groups. All experiments were conducted in triplicate. *p < 0.05, **p < 0.01, ***p < 0.001 (between groups)

We next investigated whether circ_0067557 regulates OTUB2 expression by recruiting BHLHE40. RNA pull-down assays showed that circ_0067557 markedly enriched BHLHE40 protein (Fig. 5A). Similarly, RIP assays further demonstrated significant enrichment of circ_0067557 in the BHLHE40 immunoprecipitation complex (Fig. 5B), confirming a direct interaction between circ_0067557 and BHLHE40. To determine whether this interaction affects OTUB2 transcriptional activity, a dual-luciferase reporter assay was performed. Compared with the NF-exos group, the CAF-exos group displayed significantly increased OTUB2 promoter activity, whereas circ_0067557 knockdown markedly reduced this effect in the CAF-exos + si-circ_0067557 group (Fig. 5C). FISH and immunofluorescence assays confirmed the colocalization of circ_0067557 and BHLHE40 in HCT116 cells (Fig. 5D).

To further assess the downstream role of BHLHE40, CAF-exos-treated HCT116 cells were divided into two groups, CAF-exos + sh-NC and CAF-exos + sh-BHLHE40 (workflow shown in Fig. 5E). qRT-PCR and WB analyses showed that silencing BHLHE40 significantly reduced both mRNA and protein levels of OTUB2 compared with the CAF-exos + sh-NC group (Fig. 5F-G). Combined with the previous observation that circ_0067557 upregulation enhanced OTUB2 expression without altering BHLHE40 abundance, whereas circ_0067557 knockdown reversed this effect, these findings support the conclusion that circ_0067557 promotes OTUB2 expression by recruiting BHLHE40 rather than by changing its expression level (Fig. 3I–J).

Functional analyses further showed that BHLHE40 knockdown markedly suppressed the malignant phenotypes induced by CAF-exos. Compared with the CAF-exos + sh-NC group, the CAF-exos + sh-BHLHE40 group exhibited reduced cell growth, colony formation, motility, and invasiveness, as determined by CCK-8, colony formation, Transwell, and wound healing assays. Consistently, immunofluorescence and WB analyses showed that the expression of EMT-related proteins was markedly decreased after BHLHE40 silencing. No significant difference in actin filament staining was observed between the two groups (Fig. 5H-L). These findings indicate that circ_0067557 promotes OTUB2 expression by recruiting BHLHE40, thereby enhancing EMT in CRC cells.

### CAF-exo circ_0067557 regulates tumor growth and metastasis in CRC via the BHLHE40/OTUB2 axis

**Fig. 6:**
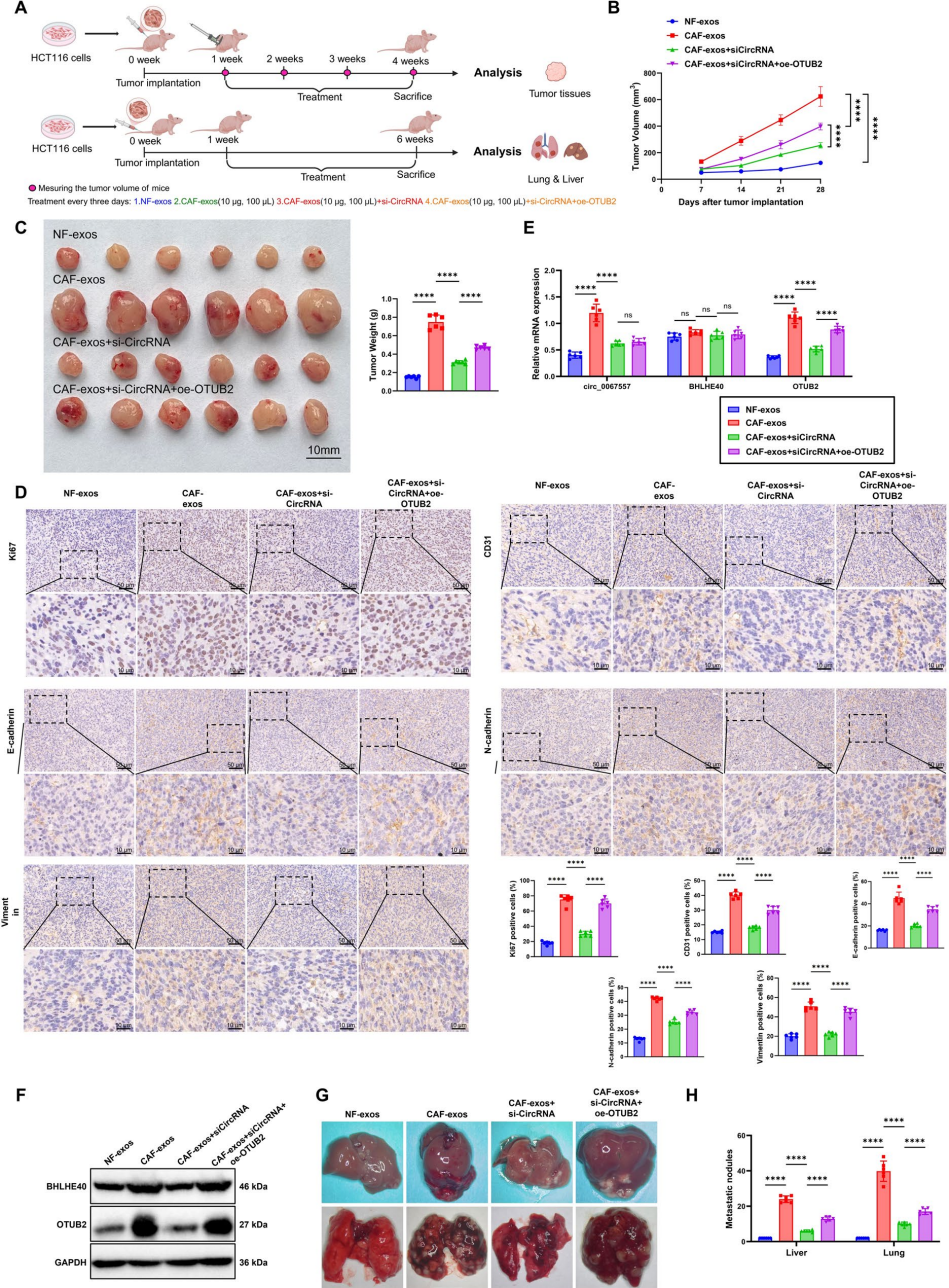
CAF-exo circ_0067557 promotes tumor growth and distant metastasis in CRC mice via the BHLHE40/OTUB2 axis. Note: (**A**) Schematic diagram of the in vivo experimental design. HCT116 cells were used to establish subcutaneous xenograft and tail vein metastasis models. Experimental groups included: NF-exos, CAF-exos, CAF-exos + si-CircRNA, and CAF-exos + si-CircRNA + oe-OTUB2; (**B**-**C**) Tumor volume was measured weekly, and tumor weight was assessed at the experimental endpoint; (**D**) Immunohistochemical staining of tumor tissues for Ki67, CD31, E-cadherin, N-cadherin, and Vimentin, accompanied by quantitative statistical analysis, bar = 50 μm; (**E**) qRT-PCR analysis of circ_0067557, BHLHE40, and OTUB2 expression in tumor tissues from each group; (**F**) WB analysis of BHLHE40 and OTUB2 protein levels in tumor tissues; (**G**) Gross images and quantification of visible metastatic nodules in the liver and lungs of each group; (**H**) Quantification of microscopic metastatic lesions in the lungs and liver to assess distant metastatic potential. Each group included six mice. *p < 0.05, **p < 0.01, ***p < 0.001 (between groups)

To evaluate the in vivo effects of CAF-exosomal circ_0067557 on CRC progression, subcutaneous xenograft and tail vein metastasis models were established using HCT116 cells. For the xenograft model, tumor growth was monitored by weekly measurement of tumor volume, and tumors were harvested at the experimental endpoint. For the metastasis model, HCT116 cells were injected through the tail vein, and lung and liver tissues were collected 6 weeks later to assess metastatic burden. Both models included four groups: NF-exos, CAF-exos, CAF-exos + si-circ_0067557, and CAF-exos + si-circ_0067557 + oe-OTUB2 (Fig. 6A).

Compared with the NF-exos group, mice treated with CAF-exos developed significantly larger and heavier tumors. Silencing circ_0067557 markedly suppressed this tumor-promoting effect, whereas OTUB2 overexpression partially restored tumor growth in the CAF-exos + si-circ_0067557 + oe-OTUB2 group (Fig. 6B-C). Immunohistochemical analysis further showed that the expression of Ki67, CD31, and EMT-related markers was markedly increased in the CAF-exos group compared with the NF-exos group. In contrast, circ_0067557 knockdown reduced the expression of these proliferation, angiogenesis, and EMT markers, whereas OTUB2 overexpression reversed these inhibitory effects (Fig. 6D). To further validate the molecular mechanism in vivo, qRT-PCR and WB analyses were performed on tumor tissues. Compared with the NF-exos group, the CAF-exos group exhibited significantly elevated expression of circ_0067557 and OTUB2. Silencing circ_0067557 markedly reduced OTUB2 expression, whereas reintroduction of OTUB2 restored its level without affecting circ_0067557 abundance. Notably, BHLHE40 expression remained unchanged among all groups at both the mRNA and protein levels (Fig. 6E, F), further supporting the conclusion that circ_0067557 regulates OTUB2 through modulation of BHLHE40 activity rather than expression.

In the tail vein metastasis model, CAF-exos markedly increased metastatic colonization compared with NF-exos, as evidenced by a greater number of visible hepatic metastatic lesions. In contrast, circ_0067557 knockdown significantly reduced the number of micrometastatic foci in both the lungs and liver. This suppressive effect was partially reversed by OTUB2 overexpression, which increased metastatic lesion numbers in both organs relative to the CAF-exos + si-circ_0067557 group (Fig. 6G-H).

Taken together, these findings demonstrate that CAF-derived exosomal circ_0067557 promotes CRC tumor growth and distant metastasis in vivo through the BHLHE40/OTUB2 axis.

## Discussion

Recent studies have emphasized the critical role of the TME on CRC metastasis [35, 36], particularly the involvement of CAFs in regulating tumor cell migration, invasion, and EMT [37, 38]. Although CAFs are known to regulate malignant behavior through cytokine secretion and extracellular matrix remodeling, the functional significance of non-coding RNAs, especially circRNAs, carried by CAF-derived exosomes remains largely unclear [14, 39]. In this study, we isolated CAF-exos from clinical CRC tissues and identified circ_0067557 as an enriched component that significantly promotes CRC cell growth, invasion, and EMT. Unlike most previous studies that focused on soluble factors or conventional exosomal components, our work specifically highlights the functional role of exosomal circRNAs and reveals a distinct mechanism by which CAFs drive tumor progression through intercellular RNA transfer.

circRNAs, known for their structural stability and highly specific expression patterns, have recently emerged as key regulators in tumor biology [40, 41]. Most previous studies have primarily described circRNAs as competitive endogenous RNAs that regulate gene expression by sponging microRNAs [42]. In contrast, our results support a miRNA-independent mechanism in which circ_0067557 directly interacts with the transcription factor BHLHE40 and enhances its transcriptional activity, representing a less explored mode of circRNA function. HCT116 cells were selected as the main experimental model because they are widely used in mechanistic studies of CRC and are supported by extensive genomic and transcriptomic datasets [43–45]. Compared with LOVO and SW480 cells, HCT116 cells exhibited relatively lower endogenous BHLHE40 expression, which reduced background interference from basal transcription factor activity and facilitated clearer evaluation of circ_0067557-dependent regulation. Our data demonstrate that circ_0067557 promotes the interaction between BHLHE40 and the OTUB2 promoter, thereby driving OTUB2 transcription and activating downstream EMT pathways. These findings broaden the recognized functions of circRNAs and underscore their direct role in transcriptional regulation.

As a basic helix-loop-helix transcription factor, BHLHE40 has been implicated in promoting EMT and tumor progression across various cancers [24, 46]. Previous studies have shown that BHLHE40 can regulate EMT-associated transcription factors such as ZEB1 and Twist and participate in EMT-related signaling pathways, whereas its upstream regulatory mechanisms remain poorly defined [47, 48]. To date, no studies have reported direct interactions between BHLHE40 and circRNAs. In the present study, ChIP-qPCR and RIP assays demonstrated that circ_0067557 specifically interacts with BHLHE40 and enhances its transcriptional activity. This finding reveals a previously unrecognized regulatory mode of BHLHE40 and expands the known interaction landscape between circRNAs and transcription factors. Emerging evidence indicates that circRNAs can regulate post-translational modifications (PTMs), including ubiquitination and phosphorylation, by interacting with ubiquitin ligases or kinases, thereby affecting protein stability and activity [49]. According to the iPTMnet database, BHLHE40 (UniProt ID: O14503) contains multiple predicted PTM sites, including ubiquitination sites at K84, K105, K117, K159, K167, and K217, as well as phosphorylation sites at S192, S207, S212, S235, and S243. These residues may provide a structural basis for post-translational regulation. Based on this, we hypothesize that circ_0067557 may regulate BHLHE40 at the post-translational level without affecting its total expression levels.

Although OTUB2 has been reported to function as a deubiquitinating enzyme involved in cell-cycle regulation and DNA damage repair, and has also been implicated in colon cancer progression through GINS1-mediated enhancement of PKM2 activity and glycolysis [50–53], its specific role in promoting EMT in CRC has remained insufficiently explored. In the present study, OTUB2 was identified as a key downstream effector of the circ_0067557–BHLHE40 axis. Our findings showed that circ_0067557 strongly regulated OTUB2 expression and that OTUB2 upregulation was accompanied by increased expression of EMT-related proteins. These results suggest that the function of OTUB2 in CRC extends beyond its canonical enzymatic activity and support its potential role as an important driver of metastatic progression. The circ_0067557–BHLHE40–OTUB2 axis identified here therefore provides new insight into the mechanism by which OTUB2 contributes to tumor dissemination.

Many current studies on circRNAs remain limited to in vitro experiments. In contrast, the present study combined subcutaneous xenograft and tail vein metastasis models to validate the tumor-promoting and metastasis-enhancing effects of circ_0067557 in vivo. The results showed that elevated circ_0067557 not only accelerated tumor growth but also markedly increased metastatic burden in distant organs, consistent with the EMT-associated phenotypes observed in vitro. These in vivo findings strengthen the biological relevance of our study and provide further support for the potential clinical translation of circRNA-based therapeutic strategies, identifying circ_0067557 as a promising target for CRC intervention.

From a translational perspective, circ_0067557 is highly enriched and stable in CAF-derived exosomes and is closely associated with CRC metastasis, suggesting its potential value as a non-invasive biomarker for identifying patients at high risk of metastatic progression. In addition, the close association of BHLHE40 and OTUB2 with metastatic potential indicates that these molecules may also contribute to predictive models for CRC metastasis. Importantly, the development of small-molecule inhibitors or antisense oligonucleotides (ASOs) targeting the interaction between circ_0067557 and BHLHE40 may offer a novel strategy to disrupt this transcriptional regulatory axis, advancing precision therapy for CRC.

Furthermore, this study enriches the current understanding of TME regulatory mechanisms by proposing a novel model in which CAFs modulate the transcriptional state of cancer cells via exosomal circRNAs. Circ_0067557 not only functions as a signaling molecule transferred to cancer cells but also induces phenotypic changes through transcriptional regulation. This finding suggests that intercellular communication between the tumor stroma and epithelial cells extends beyond traditional ligand-receptor interactions and includes nuclear-level regulatory pathways capable of driving profound transcriptional reprogramming. Such a regulatory mechanism may also be prevalent in other tumor types and requires further investigation.

Taken together, our findings identify a novel circ_0067557–BHLHE40–OTUB2 regulatory axis involved in CRC progression and provide further evidence that CAF-derived exosomal circRNAs participate in tumor-promoting communication within the TME. These results broaden the current understanding of circRNA-mediated transcriptional regulation and suggest that circ_0067557 may represent a promising biomarker and therapeutic target in metastatic CRC. Several limitations should nevertheless be noted. The sample size was limited, and other downstream pathways potentially regulated by circ_0067557 were not fully explored. In our previous study, the circ_0067557/Lin28A/Lin28B axis was also shown to promote malignant phenotypes in CRC, indicating that circ_0067557 may exert multifaceted oncogenic effects [27]. Whether these two pathways function independently, cooperatively, or hierarchically in CRC progression warrants further investigation. Future studies incorporating larger clinical cohorts, multi-omics profiling, and single-cell analyses will be needed to comprehensively characterize the regulatory landscape of circ_0067557 and to facilitate the development of more precise therapeutic strategies for CRC.

## Conclusion

This study identifies a pivotal role for CAF-exo circ_0067557 in promoting EMT in CRC. Mechanistically, circ_0067557 is transferred into CRC cells via CAF-derived exosomes, interacts with BHLHE40, enhances its binding to the OTUB2 promoter, and thereby activates OTUB2 transcription. Upregulated OTUB2 subsequently drives tumor growth, migration, invasion, and EMT. In vivo, circ_0067557 further promotes tumor growth and distant metastasis through the BHLHE40/OTUB2 axis. Collectively, these findings establish a novel CAF-exos/circ_0067557/BHLHE40/OTUB2 signaling pathway and expand current understanding of how the tumor microenvironment regulates CRC progression.

## Electronic Supplementary Material

Below is the link to the electronic supplementary material.

Supplementary Material 1: Figure S1: Expression of CAF Markers and Mesenchymal Markers in CAFs and NFs. Note: (A) Morphological observation of isolated CAFs and NFs under a microscope. Scale bar: 100 μm; (B) qRT-PCR analysis of mRNA expression levels of α-SMA, FAP, FSP-1, and Vimentin in CAFs and NFs; (C) WB analysis of protein expression levels of α-SMA, FAP, FSP-1, and Vimentin in CAFs and NFs; (D) Immunofluorescence staining for the expression and localization of α-SMA, FAP, FSP-1, and Vimentin in CAFs and NFs. Scale bar: 25 μm. All cell-based experiments were performed in triplicate. *p < 0.05, **p < 0.01, ***p < 0.001 (between groups).

Supplementary Material 2: Figure S2. Efficiency Analysis of CAF-Exos and NF-Exos Uptake by CRC Cells. Note: (A) LSCM observation of PKH67-labeled CAF-exos and NF-exos internalized by SW480 and LoVo cells. Scale bar: 25 μm; (B) Statistical analysis of PKH67-positive cell percentages in SW480, HCT116, and LoVo cells at 6, 12, and 24 h to evaluate the uptake efficiency of CAF-exos and NF-exos. All cell-based experiments were conducted in triplicate. *p < 0.05, **p < 0.01, ***p < 0.001 (between groups).

Supplementary Material 3

## Data Availability

All data generated or analyzed during this study are included in this article and/or its supplementary material files. Further enquiries can be directed to the corresponding author.

## Abbreviations

ANOVA: Analysis of Variance
BP: Biological Process
CAF: Cancer-Associated Fibroblasts
CAF-CM: Cancer-Associated Fibroblast-Conditioned Medium
CAF-Exo: Cancer-Associated Fibroblasts-Derived Exosomal
CC: Cellular Component
ceRNAs: Competitive Endogenous RNAs
ChIP: Chromatin Immunoprecipitation
ChIP-qPCR: Chromatin Immunoprecipitation Followed by qPCR
circRNA: Circular RNA
CRC: Colorectal Cancer
EMT: Epithelial-Mesenchymal Transition
FAP: Fibroblast Activation Protein
FBS: Fetal Bovine Serum
FSP-1: Fibroblast-Specific Protein 1
GO: Gene Ontology
KEGG: Kyoto Encyclopedia of Genes and Genomes
LSCM: Laser Scanning Confocal Microscopy
MF: Molecular Function
NF-CM: Normal Fibroblast-Conditioned Medium
NFs: Normal Fibroblasts
PBS: Phosphate-Buffered Saline
RIP: RNA Immunoprecipitation
SD: Standard Deviation
TME: Tumor Microenvironment
WB: Western Blot
α-SMA: α-Smooth Muscle Actin
