# Supplementary figures and images for: Cancer-associated fibroblasts-derived exosomal circ_0067557 promotes colorectal cancer epithelial-mesenchymal transition via BHLHE40-mediated transcriptional activation of OTUB2
Source: Biol Direct. 2026 May 11;21:119. doi: 10.1186/s13062-026-00805-4 (PMC13335148; doi:10.1186/s13062-026-00805-4)

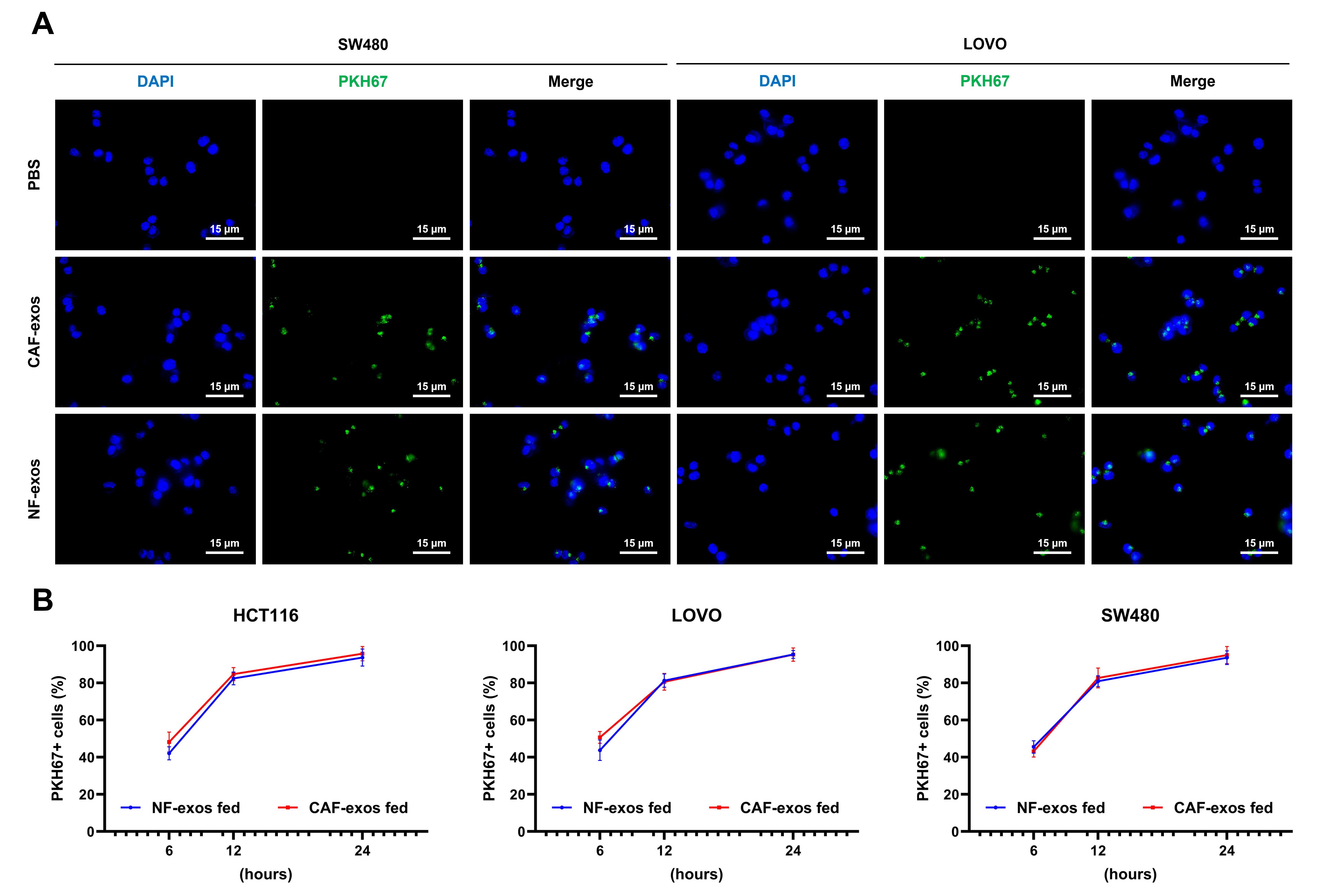

Supplement: Supplementary file 2 — Supplementary Material 2: Figure S2. Efficiency Analysis of CAF-Exos and NF-Exos Uptake by CRC Cells. Note: (A) LSCM observation of PKH67-labeled CAF-exos and NF-exos internalized by SW480 and LoVo cells. Scale bar: 25 μm; (B) Statistical analysis of PKH67-positive cell percentages in SW480, HCT116, and LoVo cells at 6, 12, and 24 h to evaluate the uptake efficiency of CAF-exos and NF-exos. All cell-based experiments were conducted in triplicate. *p < 0.05, **p < 0.01, ***p < 0.001 (between groups). [file 13062_2026_805_MOESM2_ESM.jpg]
