# Supplementary material for: Cancer-associated fibroblasts-derived exosomal circ_0067557 promotes colorectal cancer epithelial-mesenchymal transition via BHLHE40-mediated transcriptional activation of OTUB2
Source: Biol Direct. 2026 May 11;21:119. doi: 10.1186/s13062-026-00805-4 (PMC13335148; doi:10.1186/s13062-026-00805-4)
Supplement: Supplementary file 3 — Supplementary Material 3 [file 13062_2026_805_MOESM3_ESM.docx]

**Table S1. RT-qPCR primer sequences.**

| **Gene** | **Primer Sequence** |
| --- | --- |
| hsa_circ_0067557 | F: 5'- AAGTGATCTGTGTTCAAAACG -3' |
|  | R: 5'- CCTGCTGATTCCTTGGGACAA -3' |
| BHLHE40 | F: 5'- TCTAGGGGATCCTGCTGCTT-3’ |
|  | R: 5'- CTGGTGAGTCACGTCGACAA -3' |
| OTUB2 | F: 5'- TGCACTCACGAAGTAGAGCC -3' |
|  | R: 5'- TGAAGAGCCGGAATGTTCCAT -3' |
| α-SMA | F: 5'- AAAAGACAGCTACGTGGGTGA -3’ |
|  | R: 5'- GCCATGTTCTATCGGGTACTTC -3' |
| FAP | F: 5'- ATGAGCTTCCTCGTCCAATTCA -3’ |
|  | R: 5'- AGACCACCAGAGAGCATATTTTG -3' |
| FSP-1 | F: 5'- AATGTGACTCTTTGCGGGGT -3’ |
|  | R: 5'- CTCCCCATTCCCAGCATCAG -3' |
| Vimentin | F: 5'- GACGCCATCAACACCGAGTT -3’ |
|  | R: 5'- CTTTGTCGTTGGTTAGCTGGT -3' |
| GAPDH | F: 5'- CCACATCGCTCAGACACCAT -3' |
|  | R: 5'- ACCAGGCGCCCAATACG -3' |

Note: F: Forward, R: Reverse.
